# Immunometabolism Modulation in Therapy

**DOI:** 10.3390/biomedicines9070798

**Published:** 2021-07-09

**Authors:** Ezequiel Monferrer, Sabina Sanegre, Isaac Vieco-Martí, Amparo López-Carrasco, Fernando Fariñas, Antonio Villatoro, Sergio Abanades, Santos Mañes, Luis de la Cruz-Merino, Rosa Noguera, Tomás Álvaro Naranjo

**Affiliations:** 1 Department of Pathology, Medical School, University of Valencia–INCLIVA Biomedical Health Research Institute, 46010 Valencia, Spain; emonferrer@incliva.es (E.M.); sabina.sanegre@gmail.com (S.S.); iviemar@alumni.uv.es (I.V.-M.); amparolopezcarrasco@gmail.com (A.L.-C.); 2 Low Prevalenc Tumors, Centro de Investigación Biomédica En Red de Cáncer (CIBERONC), Instituto de Salud Carlos III, 28029 Madrid, Spain; 3 Ynmun Group, Institute of Clinical Immunology and Infectious Diseases, 290015 Málaga, Spain; farinas.inmunologia@gmail.com; 4 Ynmun Group, Institute of Clinical Immunology and Cell Therapy (IMMUNESTEM), 29018 Málaga, Spain; ajvillatoro@immunestem.com; 5 Integrative and Conscious Health Institute, 08008 Barcelona, Spain; s.abanades@gmail.com; 6 Centro Nacional de Biotecnología (CNB/CSIC), Department of Immunology and Oncology, 28015 Madrid, Spain; smanes@cnb.csic.es; 7 Clinical Oncology Department, Hospital Universitario Virgen Macarena, 41009 Sevilla, Spain; luis.cruz.sspa@juntadeandalucia.es; 8 Department of Pathology, Verge de la Cinta Hospital of Tortosa, Catalan Institute of Health, Institut d’Investigació Sanitària Pere Virgili (IISPV), 43500 Tortosa, Spain; 9 Department of Basic Medical Sciences, Medical School, Rovira i Virgili University, 43201 Reus, Spain

**Keywords:** metabolic reprogramming, immunometabolites, oncometabolites, regulatory balance

## Abstract

The study of cancer biology should be based around a comprehensive vision of the entire tumor ecosystem, considering the functional, bioenergetic and metabolic state of tumor cells and those of their microenvironment, and placing particular importance on immune system cells. Enhanced understanding of the molecular bases that give rise to alterations of pathways related to tumor development can open up new therapeutic intervention opportunities, such as metabolic regulation applied to immunotherapy. This review outlines the role of various oncometabolites and immunometabolites, such as TCA intermediates, in shaping pro/anti-inflammatory activity of immune cells such as MDSCs, T lymphocytes, TAMs and DCs in cancer. We also discuss the extraordinary plasticity of the immune response and its implication in immunotherapy efficacy, and highlight different therapeutic intervention possibilities based on controlling the balanced systems of specific metabolites with antagonistic functions.

## 1. Introduction

The metabolic approach in cancer biology examines the structure and metabolic pathways of the tumor ecosystem, encompassing both tumor cellularity and microenvironment (TME). The functional and bioenergetic characteristics of tumors depend on their alterations, which are linked to tumor etiopathogenesis, initial development, progression and metastasis. Specifically, tumor cells share metabolic pathways with immune cells [1], establishing resource competition systems, regulating tumor progression, immune response polarization and sensitivity to oncological treatments [2]. Therefore, therapeutic approaches from the metabolic point of view consider different intervention opportunities based on host immune response, metabolic remodeling of immune cell infiltration, tumor cellularity and other TME elements. Specifically, different tumor metabolites (immunometabolites and oncometabolites) participate in the humoral and cellular immune response, establishing the pro/anti-inflammatory balance, determining the activation of different immune cell subpopulations and regulating immune checkpoint mechanisms. Consequently, metabolic reprogramming of the tumor immune response has acquired special importance in immunotherapy approaches.

Immune system plasticity and capacity for metabolic reprogramming [3] opens up a vast field of research on the metabolic pathways involved in its physiology and pathology, an emerging area of immunometabolism knowledge. The metabolites produced by immune cells, known as immunometabolites [4] determine cell phenotype and function, act as cofactors of metabolic enzymes and mediate post-translational modifications [5]. Immunometabolites generated inside the cell can be released, acting on their surrounding environment like cytosines or at a systemic level, regulating different pro/anti-inflammatory mechanisms. Furthermore, immunometabolites influence disease progression and treatment response, thus constituting an emerging focus of interest and knowledge [5].

Tricarboxylic acid (TCA) cycle intermediate metabolites (TCAis) (acting as oncometabolites in cancer) can trigger processes of programmed cell death, autophagy, inflammation and immune signaling in response to cellular stress, pathogens, toxins or cancer [6]. TCAis are produced in the mitochondria and are distributed within the mitochondrial membranes due to their polarity and electrophilic properties. Under physiological conditions, they exert their function inside the mitochondria and can be released in a controlled way outside the cells [7]. Thus, TCAis such as succinate, fumarate, itaconate, 2-hydroxyglutarate (D-2-HG and L-2-HG) and acetyl-CoA express a wide range of non-metabolic signaling functions in physiological and pathological immunological contexts [8], especially activating the innate immune response on myeloid cells [9,10]. In turn, activation of these immune cells regulates the TCA cycle through an immune Warburg effect. In addition, TCAis such as succinate, fumarate, itaconate, citrate and α-ketoglutarate (α-KG) can also regulate activation of the inflammatory process [11] through epigenetic mechanisms [12], reactive oxygen species (ROS) modulation or post-translational modification of other proteins.

This review is focused on the regulatory metabolic pathways of both immune and tumor cells, the metabolic differences between stromal and tumor cells and especially the effector and immunosuppressive metabolic pathways of T effectors (T_eff_) and memory lymphocytes, M1 and M2 macrophages and myeloid-derived suppressor cells (MDSCs), as well as the role of acidosis and hypoxia in all of these factors. This approach consolidates the hypothesis of cancer as an energy dysfunction [13], where alterations can be found in the metabolic pathways of fatty acids (FAs), amino acids, nucleic acids and carbohydrates that affect immune dysfunction, allowing clinical tumor development. We conclude by discussing the extraordinary plasticity of the immune response, its role in immunotherapy efficacy and measures to modulate energy metabolism that should be implemented in cancer treatment.

## 2. Metabolic Pathways of the Tumor Ecosystem

### 2.1. Metabolic Reprogramming

The most widely known metabolic reprogramming process in cancer cells is the Warburg effect [14]. Compared to normal cells, tumor cells prefer using glycolysis rather than the mitochondrial oxidative phosphorylation system (OXPHOS), even in oxygen abundance states [15]. Although the glycolysis efficiency of ATP production is low, its yield is much faster, providing energy to tumor cells for growth and proliferation. Furthermore, glycolysis allows tumor cells to obtain several building blocks for biomass synthesis [16]. The oncogene *c-MYC* and hypoxia inducible factor-1 (HIF-1α) activate the expression of key enzymes which enhance aerobic glycolysis, the most important of which are glucose transporter 1 (GLUT1), hexokinase 2 (HK2), pyruvate kinase 2 (PKM2) and lactate dehydrogenase A (LDHA) [3,15,16]. GLUT1 overexpression increases glucose uptake by tumor cells. HK2 overexpression transforms glucose into glucose-6-phosphate (p), the first step in glycolysis, and enhances its flow to the pentose phosphate pathway (PPP), generating nicotinamide adenine dinucleotide phosphate oxidase (NADPH). NADPH is essential to anaerobic processes such as nucleotide synthesis, and also to protect the cell against ROS [15,16].

The regulation of pyruvate metabolism is central in the oncogenic metabolic program since this metabolite is at the crossroads between OXPHOS and lactic acid fermentation. Many cancer cells upregulate the expression of the less active PKM2 isoform, which slows down pyruvate synthesis and permits the diversion of glycolytic intermediates to other anabolic pathways, such as the serine synthesis pathway (SSP), further activating PKM2 [17]. In addition, cancer cells also downregulate the mitochondrial pyruvate carrier (MPC) complex, in charge of transporting pyruvate from the cytosol into the mitochondrial matrix. Reduced expression of MPC subunits causes the accumulation of pyruvate in the cytosol, thus favoring its conversion to lactate by lactate dehydrogenase (LDHA) [18]. Lactate is released to the TME via monocarboxylate transporter (MCT), and can be used as fuel by the cells. This is called the lactate shuttle, in which lactate is a linking vehicle between glycolytic and oxidative metabolism [19]. SSP is enhanced by the oncogene *c-MYC* which mediates the overexpression of several enzymes involved in this metabolic pathway [16,20]. Serine is the precursor for glycine, which itself is a precursor of glutathione. Therefore, tumor cells use SSP to increase glutathione and protect themselves against ROS. Serine is also necessary to initiate the one carbon metabolism (folate cycle and methionine synthesis pathway) essential for synthesis of nucleotides and many other biomolecules [20,21].

Turning to amino acid metabolic reprogramming, glutaminolysis is enhanced in many cancers [15] since cells can use glutamine as a glucose alternative to obtain energy, and it can also be converted into glutamate and then α-KG. The former is obtained in glutamine lysis by glutaminase and is necessary for nucleotide production and glutathione synthesis [22]. The latter, α-KG, is a TCA metabolite obtained from glutamate in the mitochondria via different pathways [23]. α-KG is considered to be an oncometabolite since it can be used to obtain energy, as well as in amino acid and lipid synthesis [15,16].

Lipid pathways are also involved in tumor cell metabolic reprogramming. Sterol regulatory element-binding transcription factor 1 (SREBP1), which upregulates the transcription of many enzymes of the FA synthesis pathway, is overexpressed in several cancers and has an important role in cell survival [15,16]. FA biosynthesis is frequently increased in cancer cells to meet lipid requirements for membrane synthesis, although in many cancer types fatty acid oxidation (FAO) is also enhanced in the mitochondria. The acetyl-CoA carboxylase generated by FAO enters into the TCA cycle for citrate synthesis and ATP energy can be produced by the electron transport chain [15].

Mitochondria therefore connects many essential metabolic pathways such as the folate cycle, glutamine lysis and FAO. Consequently, tumor cells increase mitochondrial biogenesis through the *c-MYC* oncogene, which transactivates mitochondrial transcription factor A [15]. Lastly, tumor metabolic reprogramming modifies the TME, regulating the immune response alongside the metabolic reprogramming capacity of immune cells themselves, and thus determining the extent of tumor progression and aggressiveness.

### 2.2. Metabolic Skewing Induced by Viral Infections

Different in vitro and in vivo studies show that many human intracellular viral, bacterial and protozoal pathogens can induce a Warburg-like metabolic state [24,25]. These pathogens hijack the host cell metabolism to redirect glycolysis and TCAis towards amino acid, lipid and nucleotide biosynthesis, which they need for their own nutritional and survival needs. Many intermediaries in the glycolytic pathway are significantly increased after viral infection. Some viruses induce glycolysis to aid replication [26], or induce metabolic change to counteract the ROS produced by the host during infection response [27,28].

Oncogenic viruses, such as human papillomavirus (HPV), hepatitis B and C (HBV and HCV), Epstein–Barr (EBV or HHV4), cytomegalovirus (CMV or HHV5), Kaposi’s sarcoma-associated herpesvirus (KSHV) and human herpesvirus 8 (HHV8) [29,30,31,32], can induce a Warburg-like effect or altered metabolic status in tumors. For example, human fibroblasts infected with CMV increase glucose consumption and lactate production, typical of Warburg metabolism [33,34]. Proteins formed during HBV replication are capable of manipulating the glucose, lipids and metabolism of nucleic acids, amino acids, vitamins and bile acids [35]. HBV has been shown to induce hepatocyte damage through dysregulation of aerobic glycolysis and lipid metabolism in a Warburg phenotype [36]. EBV stimulates oncogenesis and B-lymphocyte proliferation, hijacking mitochondrial metabolic pathways with a Warburg-like profile. As an example of this, in the study by Wang et al., human B lymphocytes were infected with EBV, revealing that shortly after infection EBV promoted oncogenesis by altering mitochondrial metabolism. Culture in a medium rich in galactose instead of glucose significantly affected transformation and proliferation of these cells, showing that glucose is a key carbon source in the transformation of B-lymphocyte metabolism when infected by EBV. EBV also expresses latent membrane protein 1 (LMP1), an oncoprotein that mimics cell CD40 signaling to activate multiple growth pathways [37]. Activation of B-lymphocyte proliferation by LMP1 has been shown to coincide with aerobic glycolysis induction [38]. Viruses also lead to production of proteins such as HPV E6 [39], which modulate central carbon metabolism in infected cells through inactivation or degradation of tumor suppressor genes such as *p53* [40].

Importantly, many intracellular pathogens also exert different mechanisms which can improve the stability and activity of proteins such as HIF-1α [41,42]. HIF-1α regulates aerobic glycolysis, whose role in carcinogenesis is well established [43]. HPV E6 protein increases cellular HIF-1α levels, promoting a Warburg effect [44]. HCV and HPV also can manipulate infected cell metabolism through HIF-1α activation [45,46]. Specifically, HCV infection stabilizes HIF-1α under normoxic conditions, facilitating glycolytic enzyme expression, while HCV-associated mitochondrial dysfunction promotes HIF-1α-mediated glycolytic adaptation. This provides crucial insight into the pathogenesis of chronic hepatitis C and possibly HCV-related hepatocellular carcinoma [47]. Similarly, a hypoxic TME can promote the activity and survival of intracellular pathogens. For example, hypoxia can induce EBV reactivation when HIF-1α binds to the *BZLF1* gene for the EBV primary latent-lytic switch [48].

### 2.3. Modulation by Exosomes

Exosomes have recently been shown to play a fundamental role in communication between cancer cells and TME cells, influencing cancer initiation, progression and metastasis [49]. Exosomes are endocytic nanovesicles, homogeneous in size (between 40–100 nm), which carry a variety of small molecules (cargo) essential for cell communication. Their cargo includes nucleic acids, proteins and lipids, and their double membrane encapsulation allows them to travel to tissues far away from their origin. Protected from degradation, they can stimulate specific receptors of target cells and horizontally transfer genetic material, triggering a pleiotropic effect [50].

Different microRNA (miRNA) have been isolated in the exosome cargo of different cancer cells. miRNA are non-coding RNA, strategic in the different stages of tumor development and expansion by allowing adaptation to a hostile environment. In early stages, primary tumor exosomes interact with contiguous cells, promoting epithelial transition (miR-200 family), fibroblast conversion into tumor cells (miR-1247-3p, miR-27a, miR-10b, miR-125b), extracellular matrix remodeling (miR-150, miR-23b) and immune system evasion (miR-197, miR-200, miR-203, miR-23a, miR-1246). As the tumor grows, its energy requirements increase beyond its blood supply, and it becomes more hypoxic, enhancing exosome production to promote angiogenesis (miR-9, miR-21, miR-210). This facilitates tumor cell release to the circulation (miR-1227) and spread to distant locations (miR-181c, miR-105, which alter the blood–brain barrier) and creating resistance mechanisms against different drugs (miR-21, miR-155, miR-222, miR-30a, miR-100-5p, miR-196a) [51].

Exosomes also play a strategic role in the metabolic reprogramming exerted in the TME and pre-metastatic niches. This allows cancer cells to adapt to a nutrient-deficient environment, modulating the stromal cells of the tumor niche towards profiles that favor the Warburg effect, promoting more aggressive and invasive phenotypes. For instance, exosomal miR-122 of some tumor types intervenes in glucose metabolism reprogramming by reducing its consumption in healthy cells surrounding the pre-metastatic niche, thereby favoring tumor development [52].

## 3. Immunometabolites and Oncometabolites

**Figure 1:**
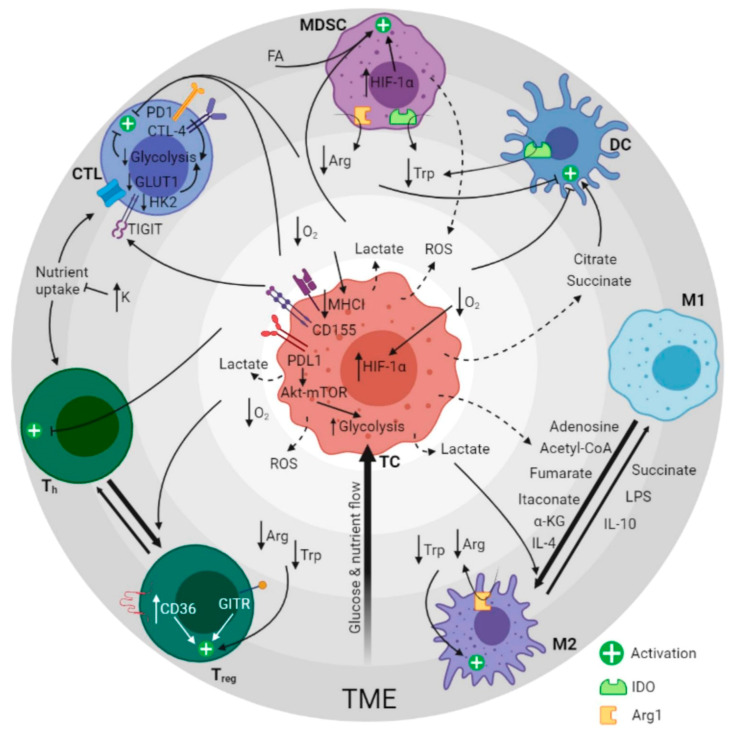
Graphical summary of the metabolic regulation of the immune response in cancer. The image depicts the crosstalking between tumor cells and immune cells by altering TME composition. Dashed arrows represent secretion of molecules, solid arrows indicate the effect of the molecules and bold arrows show immune cell type polarization direction. Concentric gray circles represent glucose and nutrient availability (dark gray = low, light gray = high) and define groups of related molecules participating synergistically in specific metabolic regulation pathways (i.e., lactate, low O_2_ and ROS). TME: tumor microenvironment. MDSC: myeloid-derived stem cells. CTL: cytotoxic T cells. Th: T helpers. Treg: T regulatory cells. M2: macrophages type 2. M1: macrophages type 1. DC: dendritic cells. FA: fatty acids. Arg: arginine. Trp: tryptophan. ROS: reactive oxygen species. α-KG: alpha-ketoglutarate. LPS: lipopolysaccharide. IDO: indoleamine-pyrrole 2,3-dioxygenase. Arg1: arginase-1. Created with BioRender.com (accessed date: 13 June 2021).

Immune cell function, activation, cytokine secretion and antitumor or antiviral effect depend on cellular metabolism [53]. Detailed knowledge of the metabolic pathways involved reveals functional differences between resting and activated immune cells, immune cells with homeostatic or altered functions, and permanent and transit TME immune cells. In general, CD8+ cytotoxic T lymphocytes (CTL), CD4+ helper T lymphocytes (T_h_), type M1 tumor-associated macrophages (TAMs) and natural killer cells play an antitumor role, while type M2 TAMs, mast cells, neutrophils and certain T- and B-lymphocyte subtypes promote cancer. However, these cells can be affected by metabolites (self-produced or immunometabolites), tumor cells or oncometabolites [54] as well as by TME conditions (Figure 1).

TCAis lead to metabolic reprogramming, which determines the functional balance of immune cells. These products possess bioenergetic, biosynthetic, immune and oncogenic actions [8] and can regulate expression of inflammatory genes [55]. In general, succinate and citrate show pro-inflammatory properties, while fumarate, itaconate and α-KG are more related to immunosuppressive functions [8]. Many of these metabolites increase during immune activation, modulating the immune activation/suppression balance. Somatic mutations in cytosolic isocitrate dehydrogenase 1 (IDH1) can lead to production of oncometabolite 2-HG, although elevated levels of 2-HG have been observed in cytogenetically normal tumors [54]. In fact, less than half the elevated 2-HG cases had IDH1 mutations, while the remaining cases had mutations in IDH2, the mitochondrial homologue of IDH1. Succinate, 2-HG and fumarate promote cancer progression, also acquiring the ability to modulate cell signaling and affect chemotherapy and radiotherapy response through epigenetic mechanisms [56,57].

Prostaglandin E2 (PGE2), produced by cyclooxygenase-2 enzyme (COX-2), is involved in anti-inflammatory cytokine generation in cancer, promoting MDSC, regulatory T lymphocyte (T_reg_) and M2 TAM accumulation. TME glucose availability is another key modulator in immune cell activation. Glucose is captured mainly by tumor cells to feed the exacerbated aerobic glycolysis (Warburg effect). This entails several phenomena, such as increased lactate production, TME acidosis and subsequent immune response regulation [58], an effect that also occurs in other, non-cancer-related inflammatory conditions [59]. Additionally, ROS produced by tumor cells participate in oxidative stress encountered by TME immune cells, while reduced blood flow in certain tumor areas results in hypoxia, which leads to HIF-1α stabilization. The HIF-1α pathway provides a metabolic switch through *c-Myc* or *Ras* oncogenes, and is therefore a critical transcriptional regulator of immunity and cancer inflammation. This metabolic reprogramming with increased glycolysis and coordinated TCA cycle rearrangement, together with reduced mitochondrial OXPHOS, enhances chronic tumor-related inflammation.

### 3.1. MDSC Fate and Function

MDSCs are heterogeneous populations of tumor-associated innate immunosuppressive cells. In MDSCs, tryptophan is involved in tumor progression. MDSCs that synthesize tryptophan-degrading enzyme indoleamine 2,3-dioxygenase (IDO) are thought to protect tumors from specific T-cell attack by inducing tolerance during the priming phase or directly in the TME through tryptophan catabolism. Moreover, MDSCs can deplete amino acids by several mechanisms, and thus determine CTL fate, growth and immune functions in the TME, such as L-arginine metabolism by arginase-1 (Arg1) activity. Besides this, MDSC-generated arginine and tryptophan depletion also facilitates TAM and T_reg_ immunosuppressive activity and hinders dendritic cell (DC) maturation.

MDSCs also sense lipid metabolites produced by the TME, which particularly enhance MDSC immunosuppressive function. In fact, some studies have suggested that tumor-associated MDSCs reprogram their metabolic pathway to adapt to a particular TME, such as one with limited O2 and glucose but high FA levels, and thus prefer to use lipids or FAs as an alternative energy source [60,61].

Nonetheless, MDSCs tend to activate their metabolism and function through aerobic glycolysis and OXPHOS [62]. The polarization of metabolism towards glycolysis generates lactate, which stimulates generation of MDSCs and phosphoenolpyruvate, an antioxidant agent that prevents ROS overproduction, contributing to MDSC survival by protecting them from apoptosis. ROS not only activate anti-oxidative pathways but also induce transcriptional programs that regulate the fate and function of MDSCs. Furthermore, MDSCs release ROS molecules as part of a major mechanism to suppress T-cell responses and modulate TAM functions, whereas hypoxia contributes to the immunosuppressive phenotype of MDSC through a mechanism linked to HIF-1α [63].

### 3.2. Complications of T-Cell Therapies

Tumor glucose uptake limits nutritional resources and IFN-γ expression in CTLs, reducing their functional response capacity [64], as also occurs in antitumor T_h_ cells [65]. PD-L1 expression may contribute to this effect by driving Akt-mTOR activation and glycolysis in cancer cells [66]. Similarly, PD-1 and CTLA-4 expression in T cells suppress aerobic glycolysis, necessary for T-cell activation [67,68], while CD155 signaling reduces T-cell glucose uptake, lactate production and GLUT1 and HK2 expression. In addition, elevated potassium levels within the TME have been recognized to disrupt T-cell nutrient uptake, leading to a stemness state, further limiting the acquisition of T_eff_ metabolism [69]. In contrast, the GITR costimulatory pathway increases T-cell proliferation and metabolic activity [70].

Among the accumulated glycolysis products generated by tumor cells, lactic acid impairs immune effector cells by directly inhibiting T-cell cytolytic functions. Likewise, high acidosis levels in a hypoxic TME result in mTOR signaling inhibition in T lymphocytes, producing energy in these cells [71,72] and promoting T_reg_ activation [73]. Interestingly, T_reg_ lymphocytes easily adapt to a lactic acid-enriched TME by CD36 upregulation [74], and are more resistant to oxidative stress-induced cell death than other T-lymphocyte subtypes [75], which would imply greater tumor tolerance in these TMEs. Taken together, these mechanisms induce T_eff_ lymphocyte inhibition and encourage tumor growth-favoring T_reg_ lymphocytes. A recent publication showed that hypoxia and glucose deprivation lead to decreased expression of major histocompatibility complex (MHC) class I molecules on tumor cells, facilitating their immune escape. Furthermore, tumor cells lose their sensitivity to IFN-γ induction, mediated by increased MHC [76]. As a consequence, tumor cells evade death by IFN-γ-producing T cells, creating another obstacle to T-cell therapies.

### 3.3. Polarization of Macrophages

Like T cells, macrophages also have a regulatory balance for their activation. The dual role of TAMs within the tumor, whether cytotoxic (pro-inflammatory, M1) or immunosuppressive (anti-inflammatory, M2), depends on the metabolic stimuli of the environment. In general, M1 metabolism usually resembles that of tumor cells, with a Warburg effect and aerobic glycolysis, while M2 metabolism tends to be based on FAO, although this might be overly simplistic [77,78]. Succinate, itaconate, fumarate and α-KG levels in macrophages and other immune cells have a profound impact on TAM polarization and innate immune memory [5,79]. Macrophage lipopolysaccharide (LPS) stimulation induces M1 macrophages, while stimulation with interleukin (IL)-4 induces M2 macrophages. TCA reprogramming in LPS-treated macrophages induces the *Irg1* gene and causes succinate and itaconate accumulation [80]. These two immunometabolites form an essential immunomodulatory system [81], succinate having an important role in inflammatory signaling [82] by IL-1β, hypoxia by HIF-1α and metabolism through ROS, while itaconate shows an anti-inflammatory role [9], inhibiting succinate dehydrogenase [83] and mediating antioxidant/anti-inflammatory pathway Nrf2 activation and modulation of IFN type I [84].

Regulation of mitochondrial respiration and FAO of TAMs are determined by their metabolic programming [85]. In TAMs, FA synthesis is considered pro-tumorigenic, unlike T_eff_, T_reg_ and T memory cells which oxidize FA for fuel.

M2 macrophages metabolize amino acids expressing high levels of Arg1, which depletes arginine and generates highly immunosuppressive polyamines [86]. Lactic acid produced by tumor cells acts as a signaler through HIF-1α and induces VEGF expression and particularly Arg1 production and M2 polarization [87]. Upregulated Arg1 in M2 TAMs also leads to immunosuppression in T cells. Mitochondrial localization of Arg2 is a central regulator of oxidative phosphorylation and macrophage polarization towards the M1 phenotype, a process controlled by miR-155 and IL-10. Arg2 increases complex II (succinate dehydrogenase) activity and downregulates succinate inflammatory mediators such as IL-10, HIF-1α and IL-1β [88].

Other mechanisms of IL-4-induced M2 TAMs regulation require the glutaminolysis-mediated production of α-KG, a cofactor of the epigenetic enzyme Jmjd3, which promotes IL-4 response in macrophages and inhibits pro-inflammatory signals [89]; this establishes a new system to balance macrophage function through the succinate/α-KG ratio. Finally, ATP citrate lyase (ACLY) of TCA cycle metabolite acetyl-CoA supports the anti-inflammatory responses of macrophages by promoting histone acetylation and IL-4-induced gene transcription [90]. ACLY is one of the main enzymes that catalyze acetyl-CoA formation; its functions include acetyl-CoA provision for lipogenesis, epigenetic regulation through histone acetylation and mediation of innate and adaptive immune responses [91].

Adenosine is a metabolite generated as a result of hypoxia inside the tumor [92] which promotes alternative macrophage activation towards M2 accumulation and expression of checkpoint inhibitors with immunosuppressive results. Furthermore, this hypoxic environment contributes to angiogenesis factor and cytosine production, also favoring accumulation of immunosuppressive M2 TAMs [93,94].

### 3.4. Dendritic Cell Subsets in Immune Response Regulation

Like macrophages, DCs undergo intense metabolic reprogramming in response to hypoxia, nutrient availability, growth factors, cytokines and other environmental signals. These include immunometabolites such as succinate and citrate [95], which regulate immunogenic/tolerogenic levels of DC. Moreover, IDO activation in DC has been shown to be involved in tumor immune evasion. Similarly, lactate produced through glycolysis can reduce DC activation and antigen presentation, facilitating tumor cell escape from immune attack [96,97].

## 4. Therapeutic Applications of Immunometabolism Regulation

**Figure 2:**
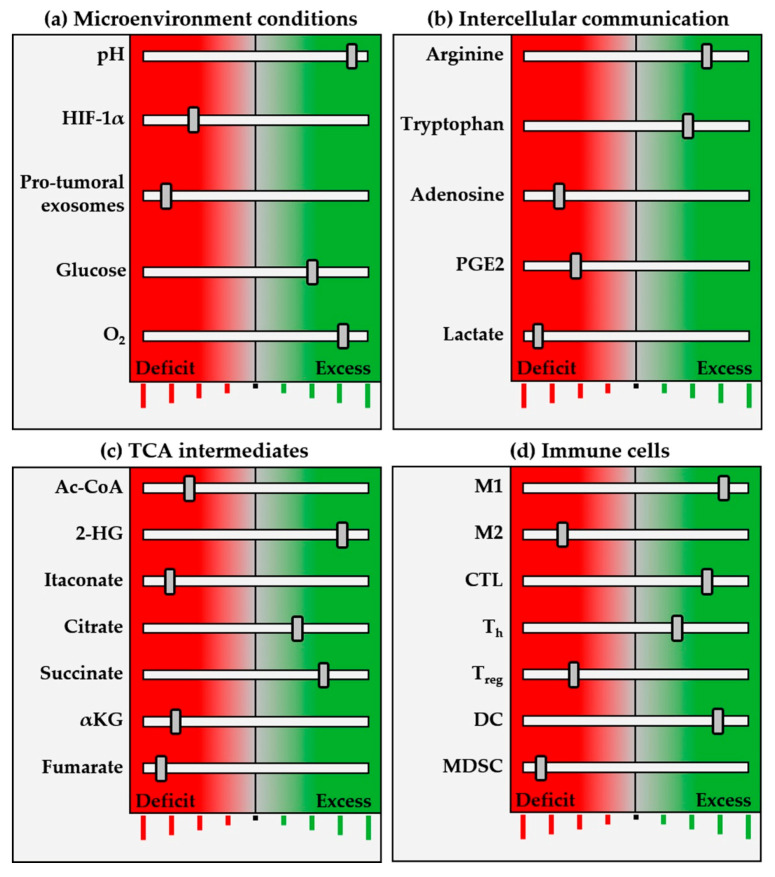
Schematic representation of pro-inflammatory (antitumor) immunometabolic and cellular profile. Immune system programming begins at the perinatal stage, matures through environmental stimulation and is regulated by a balanced network that includes genetic and epigenetic profile, microbiota, hypothalamic–pituitary–adrenal (H–P–A) axis, sleep, diet and many other elements. Within this network, metabolism amplifies or inhibits immune function, determining physiological, adaptive or altered responses by (**a**) microenvironmental conditions, such as pH, oxygenation, etc.; (**b**) intercellular communication with chemokines, lactate, PGE2, etc.; and (**c**) TCAis, such as succinate, α-KG and others. The figure shown represents different frequency (inhibition/activation) bands, with an equilibrium position in the center, decreased activity towards the left (red), and increased towards the right (green). Like an equalization system, this network selects the immune “frequency band” determined by fine-tuning the elements making up the expanded system. This determines the immune response pattern, defined by cell subpopulations (**d**), their functionality, cytokine secretion and intercellular communication relationships within the TME. This schema introduces a concept that must be investigated and determined separately for each type of immune response, depending on specific physiological or pathophysiological situations, thus providing a spectrum of frequencies in which the greater the number of immune equalizer bands, the finer the tuning and function. Accumulated knowledge in oncological biology has brought into vision an immunometabolic equalizer with pro-/anti-tumor, pro-/anti-inflammatory, hot/cold tumor and pro-/anti-therapeutic-related functions. The precise immunometabolic alterations of each patient and tumor emerge as promising biological markers to guide immunotherapy treatments. The proposed immunometabolic equalizer schema represents crosstalk between the balance of different metabolic regulation levels (**a**–**c**), capable of determining the balance between pro-/anti-inflammatory cell populations, and unblocking immune checkpoints. The equalizer is not autonomous, but is connected to an amplifier in the form of macroenvironment elements such as the H–P–A axis, microbiota, stress, diet, exercise and medications, which in turn have their own equalization controls, in a fractal-like manner.

In cancer, metabolites have an especially significant effect on immune cells, hence cancer immunotherapy aims to act at the metabolic level (Figure 2). For example, PGE2’s effect can be countered by COX-2 inhibitors such as acetylsalicylic acid or celecoxib, as established in colorectal cancer, mainly in the context of chemoprevention strategies; clinical research in this field is, however, still ongoing. Regarding 2-HG oncometabolite production, glutamine and glutamate pathways can be inhibited, providing alternative potential targets. Furthermore, the exceptional glucose metabolism of cancer cells (Warburg effect) implies TME acidification and lower glucose availability. These events seem key in explaining the wide range of immunosuppressive effects associated with cancer cell-induced biochemical reactions. Thus, regarding the metabolic switch to glycolysis mediated by metformin, dependent suppression of the mitochondrial complex followed by glucose deprivation could be used in combination with different chemotherapy schedules or immune checkpoint inhibitors as new cancer therapy approaches.

Immunometabolism therefore represents an emerging new target for cancer immunotherapy, and new lines of research are focused on directly or indirectly regulating different metabolites to improve current immunotherapeutic approaches (Table 1).

### 4.1. Immunotherapy Based on Exosomes

Tumor exosomes modify immune cell metabolism, helping the tumor evade the immune response, so different strategies against exosome biogenesis and their mechanism of action make interesting targets against cancer [51,52]. Among the alternatives are use of exosomes released by immune cells to suppress tumor proliferation, inhibiting tumor exosome production or blocking their uptake by receptor cells, as well as designing bioengineered exosomes as transporters of anti-cancer products (miRNA, siRNA, proteins, drugs or vaccine) [98]. In addition to their cargo specificity, exosomes are released in greater quantities by cancer cells than healthy cells, so they can be isolated from body fluids (liquid biopsy), giving them great potential as biomarkers.

Consequently, to date there have been around 50 clinical studies related to exosomes and cancer (www.clinicaltrials.gov, accessed on 15 June 2021), most focused on diagnostic/prognostic or drug response biomarkers, while others are centered on using exosomes as antitumor therapeutic elements [99].

### 4.2. T-Cell Regulation

Although viruses modify the immune response, antiviral responses often successfully eliminate infectious agents or keep them under control throughout life, as evidenced by the infrequent occurrence of CMV- or EBV-mediated disease in healthy individuals despite persistent infection in up to 90% of the human population [46]. This has led to the interesting proposition of repurposing antiviral T cells against tumors. In a recent study, Rosato et al. demonstrated that antiviral T cells can target tumors when loaded with exogenous viral peptide. This strategy became even more efficient when combined with checkpoint blocking [100], potentially opening up new therapeutic avenues. It remains to be determined whether such a strategy influences anti-viral control or whether use of antiviral T cells could eventually lead to their depletion and/or reprogramming to T_reg_ lymphocytes in a suppressor TME. Therefore, the choice of viral target peptides and combination with other strategies is crucial, especially considering that common persistent viruses such as EBV are oncogenic if uncontrolled [101].

Another therapeutic approach consists of intratumoral administration, through lipid nanoparticles, of an interfering RNA that silences lactate dehydrogenase A (LDH-A) production and activity [102]. In animal models of melanoma, anti-PD1 treatment has been shown to improve mice with LDH-A-deficient tumors [103]. One study also reported that deletion of LDH-A in myeloid cells can induce antitumor immunity of T lymphocytes against lung carcinoma [104]. In addition, the combination of pemetrexed (antifolate antineoplastic agent) with anti-PD-L1 shows direct antitumor effects together with improved CTL metabolism and immune function, by stimulating mitochondrial biogenesis and thus facilitating their activation and antitumor effect [105].

The metabolic differences observed between tumor cells and immune cells invite an exploration of opportunities for T-cell metabolic reprogramming prior to starting other cancer treatments, especially immunotherapy. Among the metabolic targets to improve immune response are Arg1, IDO, lactate, CD36, CD73 and D-2-HG [53,74]. The study of these and other immunometabolites creates a need to identify specific metabolic determinants of response, which help gain objective insight into the metabolic consequences of checkpoint therapy.

As an illustration, CD36 ablation in T_reg_ reduced its survival in TME conditions, leading to tumor growth suppression, antitumor activity enhancement of T cells and additive antitumor responses with anti-PD1 therapy [74]. Alternatively, one strategy to develop therapeutic agents against autoimmune diseases uses the immunometabolite 2-HG, which can epigenetically regulate the balance between pro-inflammatory T_h17_ cells and induced T_reg_ (iT_reg_) cells towards T_h17_ differentiation [106]. Increased transamination, catalyzed by GOT1, produces increased 2-HG levels in T_h17_ cells, hypermethylation of the *Foxp3* gene locus and inhibition of *Foxp3* transcription, determining T_h17_ differentiation. When glutamate−α-KG conversion is inhibited, 2-HG production decreases, methylation of the *Foxp3* gene locus is reduced and *Foxp3* expression increases, resulting in T_h17_ cell differentiation blockade and iT_reg_ cell development. Selective inhibition of GOT1 with (aminooxy) acetic acid has been shown to improve autoimmunity by regulating T_h17_/iT_reg_ fate, conforming to a regulatory system based on a 2-HG/(aminooxy) acetic acid balance.

Alternatively, considering that tumor glucose consumption restricts the glycolytic capacity and IFN-γ production of T cells, the checkpoint blockade can be mitigated with antibodies against PD-1, its ligands and CTLA-4 [66]. In addition, glucose availability in the TME can be enhanced, which allows improved CTL cytokine expression and avoids toxic concentrations of certain metabolites, such as adenosine, kynurenine and ornithine, ROS and increased acidosis, thus preventing antitumor immune response suppression. Likewise, a recent report showed that acetate could be used as an alternative carbon source and rescue the functions (such as IFN-γ production) of exhausted T lymphocytes infiltrating the tumor [107]. It has been described that high potassium reduces T-cell nutrient uptake, which results in T-cell stemness and exhaustion. Interestingly, treatment of antitumor T cells with elevated extracellular potassium, as well as pharmacologic or gene therapies, enables enhanced tumor destruction during immunotherapy performance [69]. Other actions in the TME are inhibition of the regulatory immunometabolite 1-methylnicotinamide, which is noticeably increased in tumor-infiltrating T cells [108], and expression of the enzyme catalase by CAR- (chimeric antigen receptor) T cells, through which these cells are better protected from ROS-induced oxidative stress [109].

### 4.3. Macrophage Regulation with Immunometabolites

Highly immunosuppressive phenotypes have been used as targets in immunotherapeutic treatment, with the aim of reverting their function to an immunoprotective role. MDSC enhances crosstalk with TAMs through an IL10- and cell contact-dependent mechanism, and skews them towards an M2 phenotype, leading to an immunosuppressive environment. Therefore, immunotherapies aiming to polarize TAMs into M1-like macrophages have been suggested as a therapeutic approach against cancer. MDSC function can be regulated by therapeutically altering its metabolism, as achieved with D-2-HG by blocking the glycolytic pathway [110] or using Arg1 and IDO inhibitors, thus hindering TAM−M2 polarization. Moreover, as CD73 is a cell-surface glycoprotein essential for extracellular adenosine generation, CD73 inhibition combined with other strategies induces antitumor effects in preclinical mouse models of cancer. In fact, several anti-CD73 antibodies are under clinical research in phase I–II trials, mostly combined with immune checkpoint inhibitors.

Since Nrf2 activation is necessary for the anti-inflammatory action of itaconate in TAMs, another therapeutic alternative has sought itaconate derivatives to reproduce this action at a therapeutic level, such as 4-octyl itaconate, which protects against LPS-induced lethality and decreases cytokine production [84]. Likewise, IFN type I is frequently used in immunotherapy [111], due to its ability to increase Arg1 expression and itaconate production, which forms a new negative feedback loop through the IFN/itaconate system [84] mediated by inflammatory macrophages.

## 5. Discussion

Reprogramming tumor cell metabolism allows cells to acquire growth and survival advantages, while tumor ecosystem changes in the form of acidosis, hypoxia, nutrient depletion and cellular waste accumulation affect the action of the cellular elements responsible for an effective immune response, promoting, for example, tumor immune evasion.

Different metabolism types and possible tumor and stromal cell abnormalities play key roles in shaping the degree of morphological, phenotypic and molecular heterogeneity within the tumor, and the extent of response or resistance to oncological, cytotoxic, immunotherapeutic or other treatments. This pinpoints the metabolism of tumor and immune cells as a preferred target for new research lines and treatment development, due to the characteristic metabolic plasticity of these cells. This forms the basis for intervening in immunometabolism to reverse immune escape and favor the anti-tumor immune response, an approach that involves combining metabolic inhibitors with immune checkpoint inhibitors, considering immune-regulatory metabolites on the TME as immune targets, and taking tumor and immune cell metabolism into account to improve the efficacy of immunotherapy.

Accordingly, the importance of studying TCA intermediates and other metabolites in immunotherapy arises from their regulatory role in cancer, which can be identified at various levels. At one level is the immune balance that determines the pro-inflammatory versus anti-inflammatory effect, such as T_h17_/T_reg_ regulated by 2-HG/(aminooxy) acetic acid balance, or M1/M2 TAMs regulated by succinate/itaconate, succinate/α-KG and IFN/itaconate balancing systems. At another lies energy balance, which regulates CTL and T_eff_ function via glucose availability for tumor cells versus antitumor immune cells. These regulatory systems are not exclusive to oncological pathophysiology, but are available as immune and inflammatory control systems in different pathological situations. Thus, the balance between T_h17_/T_reg_ establishes the degree and rhythm of both physiological and pathological autoimmunity. The shift from T_h17_ to T_reg_ protects against autoimmune inflammation, and this fine balance is regulated by 2-HG inhibition [106].

On another plane, itaconate, derived from the TCA cycle, is an immunometabolite with a direct antimicrobial effect, which by inhibiting isocitrate lyase reduces the production of pro-inflammatory mediators in macrophages and improves sepsis and psoriasis in animal models [112]. This action, combined with succinate, offers a balanced macrophage control system, which can be reprogrammed from a pro-inflammatory to anti-inflammatory state, with the aim of limiting the damage of the inflammatory process, facilitating tissue repair and preventing inflammatory diseases and tumors from becoming unhealable wounds.

Combinations of new metabolic modulators with immune checkpoint inhibitors are a common strategy currently in clinical research, as it seems anti PD1/PD-L1 antibodies may restore the metabolic fitness of T lymphocytes. In addition, the favorable safety profile of immune checkpoint inhibitors (particularly against PD1 and PD-L1) has paved the way for extensive testing of combined immunometabolic approaches. Unfortunately, the phase III results of the epacadostat (IDO inhibitor) plus pembrolizumab (anti-PD1/PD-L1 monoclonal antibody) combination in metastatic melanoma were disappointing, highlighting the fact that this type of interventional target is not easily modulated; they represent a major challenge requiring finely tuned approaches, preferably based on reliable biomarkers and new technical strategies selectively targeting TME.

In summary, the entire tumor ecosystem is subject to metabolic functional regulation mechanisms, which shape the polarization of the immune response, in both a cytotoxic and immunosuppressive sense, and modulating these mechanisms opens the door to complementary therapies in different pathologies, with special importance in oncology. New clinical and translational data hopefully available in the near future will elucidate the true value of the aforementioned therapies.

## Figures and Tables

**Table 1:** Therapeutic application of immunometabolism regulation. Forty studies related to immunometabolism regulation in cancer were found in clinicaltrials.gov database (accessed on 15 June 2021) using the following Boolean search string: “(immune AND reprogramming) OR (regulation AND metabolism AND immune) OR (immunotherapy AND metabolism AND modulation)”. Here are summarized 10 of the most representative current studies in the field.

Study Type	Status	Study Title	Conditions	Identifier
Interventional (Clinical Trial)	Active, not recruiting	Gene and Vaccine Therapy in Treating Patients With Advanced Malignancies	Malignant Neoplasm	NCT01697527
Interventional (Clinical Trial)	Not yet recruiting	Metformin for Chemoprevention of Lung Cancer in High Risk Obese Individuals	Lung Carcinoma	NCT04931017
Interventional (Clinical Trial)	Not yet recruiting	Microenvironment and Immunity of Digestive Cancers - East Paris Multicentric Cohort (MICADO)	Colorectal Cancer Pancreas Tumor Biliary Tract Tumor Immune System and Related Disorders	NCT04707365
Interventional (Clinical Trial)	Recruiting	Lower Dose Decitabine (DAC)-Primed TC (Carboplatin-Paclitaxel) Regimen in Ovary Cancer (DAC and CT)	Primary Malignant Neoplasm of Ovary FIGO Stages II to IV	NCT02159820
Interventional (Clinical Trial)	Recruiting	Paclitaxel + Carboplatin + Durvalumab With or Without Oleclumab for Previously Untreated Locally Recurrent Inoperable or Metastatic TNBC (SYNERGY)	Triple-Negative Breast Cancer (TNBC)	NCT03616886
Interventional (Clinical Trial)	Recruiting	Study of Sirolimus in Patients With Advanced Pancreatic Cancer	Pancreatic Cancer	NCT03662412
Observational	Active, not recruiting	Myeloid Cell Reprogramming in the Context of Radioiodine Therapy in Patients With Non-Medullary Thyroid Carcinoma	Thyroid Cancer	NCT03397238
Observational	Not yet recruiting	Targeting Potassium Channels to Reprogram Glioblastoma Microenvironment: In Vitro and In Vivo Studies	Cancer of Head and Neck	NCT03954691
Observational	Not yet recruiting	Evaluating Immunological Parameters, Neurocognitive Changes, Activity Levels, and Driving Fitness in Patients Undergoing CAR-T Cell Therapy	Hematologic Neoplasms	NCT04275154
Observational	Recruiting	The Mechanism of Enhancing the Anti-Tumor Effects of CAR-T on PC by Gut Microbiota Regulation	Pancreatic Cancer Gut Microbiota CAR-T	NCT04203459
